# Disease Severity of Respiratory Syncytial Virus Infection in Hospitalized Children

**DOI:** 10.3390/v18040451

**Published:** 2026-04-09

**Authors:** Costanza Di Chiara, Vera Rigamonti, Beatrice Rita Campana, Anna Chiara Vittucci, Livia Antilici, Flaminia Ruberti, Hajrie Seferi, Giulia Brigadoi, Daniele Donà, Alberto Villani, Anna Cantarutti, Susanna Esposito

**Affiliations:** 1 Child Health Evaluative Sciences, The Hospital for Sick Children, Toronto, ON M5G 0A4, Canada; costanza.dichiara@sickkids.ca; 2 Società Servizi Telematici—Pedianet, 35138 Padova, Italy; v.rigamonti@sosepe.com; 3 Pediatric Clinic, Department of Medicine and Surgery, Parma University Hospital, 43126 Parma, Italy; beatricerita.campana@unipr.it (B.R.C.); flaminia.ruberti@unipr.it (F.R.); hajrie.seferi@unipr.it (H.S.); susannamariaroberta.esposito@unipr.it (S.E.); 4 General Pediatric and Infectious Disease Unit, Pediatric Emergency Medicine, Bambino Gesù Children’s Hospital, IRCCS, 00165 Rome, Italy; annachiara.vittucci@opbg.net (A.C.V.); livia.antilici@opbg.net (L.A.); alberto.villani@opbg.net (A.V.); 5 Department for Women’s and Children’s Health, University of Padova, 35128 Padova, Italy; giulia.brigadoi@unipd.it (G.B.); daniele.dona@unipd.it (D.D.); 6 Systems Medicine Departments, Tor Vergata University of Rome, 00133 Rome, Italy; 7 Department of Statistic and Quantitative Methods, Division of Biostatistics, Epidemiology and Public Health, University of Milano-Bicocca, 20126 Milano, Italy

**Keywords:** respiratory syncytial virus, viral coinfection, rhinovirus, pediatric hospitalization, pediatric infectious diseases, multiplex polymerase chain reaction

## Abstract

**Background**: Respiratory syncytial virus (RSV) is a leading cause of hospitalization for acute respiratory tract infection (ARTI) in young children. Respiratory viral coinfections are frequently identified in RSV-related ARTIs, yet their impact on disease severity remains controversial and may vary according to the co-pathogen involved. In the context of evolving RSV prevention strategies, a clearer understanding of RSV coinfection phenotypes is needed. **Methods**: We conducted a multicenter retrospective cohort study of children aged ≤ 5 years hospitalized for ARTI at two Italian tertiary-care pediatric hospitals between 1 September 2022 and 30 April 2025. Children with laboratory-confirmed RSV infection detected by multiplex polymerase chain reaction were included. Patients were classified as having RSV monoinfection, RSV–rhinovirus coinfection, or RSV–non-rhinovirus coinfection. Severe disease was defined as a composite outcome including intensive care unit (ICU) admission, need for respiratory or hemodynamic support, or death. Association between infection status and severe disease was evaluated using a Poisson regression model with robust variance, adjusted for age, sex, and comorbidities. **Results**: Among 231 RSV-related hospitalizations, 118 (51.1%) were classified as RSV monoinfection, 65 (28.1%) as RSV–rhinovirus coinfection, and 48 (20.8%) as RSV–non-rhinovirus coinfection. Children with RSV–rhinovirus coinfection were older and had shorter hospital stays. Severe disease occurred in 80.5% of RSV monoinfections, 70.8% of RSV–rhinovirus coinfections, and 75.0% of RSV–non-rhinovirus coinfections. After adjustment, neither RSV–rhinovirus coinfection (adjusted risk ratio [aRR]: 0.93; 95% confidence interval [95% CI]: 0.80–1.13) nor RSV–non-rhinovirus coinfection (aRR: 0.99; 95% CI: 0.83–1.18) was associated with increased disease severity compared with RSV monoinfection. **Conclusions**: RSV–rhinovirus and RSV–non-rhinovirus coinfections were not associated with greater disease severity compared with RSV monoinfection in hospitalized children. These findings support pathogen-specific interpretation of multiplex diagnostic results and inform clinical risk stratification in the era of expanding RSV prevention strategies.

## 1. Introduction

Respiratory syncytial virus (RSV) is a major cause of morbidity and mortality in infants and young children worldwide. Clinical manifestations of RSV infection range from mild, self-limiting upper respiratory tract infection (URTI) to severe lower respiratory tract infection (LRTI) requiring hospitalization and respiratory support [1,2,3,4,5]. While young age, prematurity, low birth weight, and underlying medical conditions are well-established risk factors for severe RSV disease [2,6,7,8], the contribution of viral factors—particularly respiratory viral coinfections—remains less clearly defined.

Respiratory viral coinfections are increasingly detected among children hospitalized with RSV, with reported prevalence ranging from approximately 20% to over 40%, depending on age, geographic setting, season, and diagnostic strategy [9,10]. However, the clinical significance of RSV with respiratory viral coinfection remains controversial [10,11]. Some studies suggest that viral coinfections may exacerbate disease severity through additive or synergistic pathogenic mechanisms, whereas others report no difference—or even milder disease—compared with RSV monoinfection [10,11]. These conflicting findings likely reflect heterogeneity in study populations, outcome definitions, and, critically, the specific viral copathogens involved.

Rhinovirus is the most frequently identified copathogen in children hospitalized with RSV and circulates ubiquitously across pediatric age groups [12]. Beyond its high prevalence, rhinovirus represents a biologically relevant copathogen in the context of respiratory viral coinfections [13,14,15]. Experimental studies have shown that rhinovirus infection can induce robust interferon-mediated antiviral responses, potentially limiting replication of other respiratory viruses through mechanisms of viral interference [13,14,15]. At the same time, evidence from RSV–rhinovirus coinfections suggests that these interactions are complex and not uniformly protective, with studies reporting variable clinical outcomes ranging from similar or attenuated disease to increased severity depending on the population and study design [16,17]. More broadly, evidence from other respiratory viral coinfections, such as influenza, adenovirus, or human metapneumovirus, suggests that virus–virus interactions may be heterogeneous and context-dependent, with some combinations associated with attenuated disease and others with increased severity [8,9,10,11,12,13,14,15]. These observations highlight that the clinical impact of coinfection may depend on the specific viral pairing rather than coinfection status alone.

The epidemiology and clinical presentation of RSV disease are changing in the context of newly implemented prevention strategies. Nirsevimab, a long-acting monoclonal antibody targeting the RSV fusion protein, has shown high efficacy in preventing RSV-associated LRTI hospitalizations in both preterm and term infants [18,19]. In parallel, maternal RSV vaccination has emerged as an additional preventive strategy, providing passive immunity to infants during the first months of life [20,21]. Early real-world implementation of these approaches has already begun to alter the demographic and clinical characteristics of children hospitalized with RSV [22]. As RSV prevention strategies modify disease burden and hospitalization patterns, understanding whether viral coinfections independently contribute to disease severity has become increasingly relevant for clinical risk stratification and the interpretation of multiplex diagnostic results. In this context, we conducted a retrospective cohort study to compare disease severity among children hospitalized with RSV monoinfection and those with RSV viral coinfections. We defined a priori separate exposure groups for RSV–rhinovirus and RSV–non-rhinovirus coinfections to better capture potential pathogen-specific differences in clinical outcomes.

## 2. Materials and Methods

### 2.1. Study Design and Setting

We conducted a multicenter retrospective cohort study including children aged ≤ 5 years hospitalized for acute respiratory infection (ARTI) at Pietro Barilla Children’s Hospital (Parma, Italy) and Bambino Gesù Children’s Hospital (Rome, Italy). The study period covered three consecutive RSV seasons, from 1 September 2022 to 30 April 2025, spanning the immediate early and post-nirsevimab implementation in Italy [23].

Both participating hospitals are tertiary-care referral centers with dedicated pediatric wards and pediatric intensive care units (PICUs), serving large and heterogeneous pediatric populations. Standardized clinical pathways for the management of pediatric ARTIs are implemented at both sites.

The study was conducted in accordance with the Declaration of Helsinki and approved by the local ethics committees of the participating hospitals (Parma: AVEN 65/2025/OSS/AOUPR; Rome: 3645/2025). Given the retrospective design and use of anonymized routinely collected clinical data, the requirement for informed consent was waived.

### 2.2. Study Population

This retrospective study included children aged ≤ 5 years who were hospitalized at either participating hospital during the study period with a primary diagnosis of ARTI and who underwent respiratory multiplex polymerase chain reaction (PCR) testing for viral pathogens at admission. Only children with laboratory-confirmed RSV infection were included in the analysis. Children were excluded if multiplex PCR testing was incomplete or did not include the full viral panel required for exposure classification, or if hospitalization occurred outside the predefined study period. To avoid within-subject correlation, only the first hospitalization episode per patient during the study period was included in the analysis.

### 2.3. Virological Testing and Exposure Definition

Respiratory samples (nasopharyngeal swabs or aspirates) were collected as part of routine clinical care and analyzed using commercially available multiplex PCR assays capable of detecting RSV and a broad range of respiratory viruses, including enterovirus/rhinovirus, influenza A and B viruses, parainfluenza viruses, adenovirus, human metapneumovirus, and seasonal coronaviruses. Based on multiplex PCR results, patients were classified into three mutually exclusive exposure groups: (i) RSV monoinfection, defined as detection of RSV alone; (ii) RSV–rhinovirus coinfection, defined as simultaneous detection of RSV and rhinovirus; (iii) RSV–non-rhinovirus coinfection, defined as detection of RSV plus one or more respiratory viruses other than rhinovirus. This categorization was defined a priori to distinguish rhinovirus coinfection from other viral combinations, given evidence suggesting differential clinical effects associated with RSV–rhinovirus interactions.

### 2.4. Covariates and Clinical Data Collection

Demographic and baseline clinical data were retrospectively extracted from electronic medical records using standardized data collection forms. Collected variables included age at admission (<1 month, 1 mo.–<1 year, 1–5 yrs.), sex (female or male), ethnicity (Caucasian, African, Asian, Middle Eastern, Hispanic), perinatal characteristics (i.e., prematurity status, gestational age at birth, and birth weight), presence of underlying medical conditions (i.e., chronic respiratory, cardiac, neurological, renal, or other chronic diseases), symptoms at admission, and management.

### 2.5. Outcome Definition

The primary outcome was severe disease, defined as the occurrence of at least one of the following during hospitalization: (i) admission to the PICU; (ii) requirement for respiratory support, including supplemental oxygen, non-invasive ventilation, or invasive mechanical ventilation; (iii) requirement for hemodynamic support (e.g., vasoactive medications); or (iv) death from any cause during hospitalization. Secondary outcomes included individual components of the composite severe outcome and length of hospitalization.

### 2.6. Statistical Analysis

Descriptive statistics were used to summarize demographic and clinical characteristics by exposure groups. Categorical variables were reported as counts and percentages and compared using the chi-square test or Fisher’s exact test, as appropriate.

The association between RSV viral exposure category and severe disease was evaluated using a Poisson regression model with robust (sandwich) variance estimators, allowing direct estimation of adjust risk ratios (aRRs) and 95% confidence intervals (95% CIs). RSV monoinfection was used as the reference group. Multivariable models were adjusted a priori for clinically relevant confounders, including age, sex, and presence of comorbidities (any vs. none).

All analyses were performed using two-sided tests, with a *p*-value < 0.05 considered statistically significant. Statistical analyses were conducted using SAS version 9.4 and R version 4.5.1.

## 3. Results

### 3.1. Characteristics of the Study Population

During the study period, 579 hospitalizations for ARTI were identified across the two participating hospitals, of which 231 (22.2%) had laboratory-confirmed RSV infection and were included in the analysis, including 120 from Parma and 111 from Rome (Supplementary Figures S1 and S2).

Among the included RSV-related hospitalizations, 118 (51.1%) children were classified as RSV monoinfection, 65 (28.1%) as RSV–rhinovirus coinfection, and 48 (20.8%) as RSV–non-rhinovirus coinfection (Table 1). RSV–non-rhinovirus coinfections comprised a heterogeneous group of viral combinations, including adenovirus (*n* = 25, 52.1%), non–SARS-CoV-2 human coronavirus (22, 45.8%), influenza (11, 22.9%), parainfluenza (7, 14.6%), metapneumovirus (7, 14.6%), and SARS-CoV-2 (6, 12.5%), with multiple pathogens detected in some cases (Table 1, Supplemental Figure S3).

Sex distribution was similar across exposure groups. Age distribution differed by exposure category (*p* = 0.003), with RSV–rhinovirus coinfection more frequent among children aged 1–5 years (41.5%), whereas RSV monoinfection and RSV–non-rhinovirus coinfection predominantly affected infants aged 1 month to <1 year (78.8% and 66.7%, respectively). Ethnicity was comparable across groups, with the majority of children being Caucasian in all exposure categories. The prevalence of prematurity, low birth weight, and underlying medical conditions did not differ among children with RSV monoinfection, those with RSV–rhinovirus coinfection, and those with RSV–non-rhinovirus coinfection (Table 2).

### 3.2. Clinical Manifestations

Presenting symptoms at admission are detailed in Table 3. Fever and cough were common across all exposure groups, with no differences observed. Rhinitis was reported less frequently among children with RSV–rhinovirus coinfection compared with RSV monoinfection and RSV–non-rhinovirus coinfection (24.6% vs. 40.7% and 45.8%, respectively, *p* = 0.034). Loss of appetite tended to be less frequent among children with RSV–rhinovirus coinfection (27.7%) compared with those with RSV monoinfection and RSV–non-rhinovirus coinfection (44.1% and 45.8%, respectively), although this difference did not reach statistical significance (*p* = 0.061). Gastrointestinal symptoms were more commonly reported among children with RSV–rhinovirus and RSV–non-rhinovirus coinfections (20.0% and 16.7%) than among those with RSV monoinfection (8.5%, *p* = 0.069).

### 3.3. Clinical Outcomes

Clinical outcomes during hospitalization are summarized in Table 4. Overall, the distribution of severe outcomes was similar across exposure groups. Rates of PICU admission and need for respiratory support did not differ between children with RSV monoinfection, RSV–rhinovirus coinfection, and RSV–non-rhinovirus coinfection. Consistently, the composite severe outcome occurred at comparable frequencies across groups, suggesting that viral coinfection status was not associated with increased disease severity in this cohort.

In contrast, length of hospitalization differed between groups (*p* = 0.003). Children with RSV–rhinovirus coinfection were more likely to have shorter hospital stays (≤1 week), whereas longer hospitalizations (1 week to <1 month) were more frequent among children with RSV monoinfection and RSV–non-rhinovirus coinfection (Table 4). The RSV–non-rhinovirus coinfection group included a heterogeneous combination of respiratory viruses, and the study was not powered to assess differences according to individual viral pathogens.

**Figure 1:**
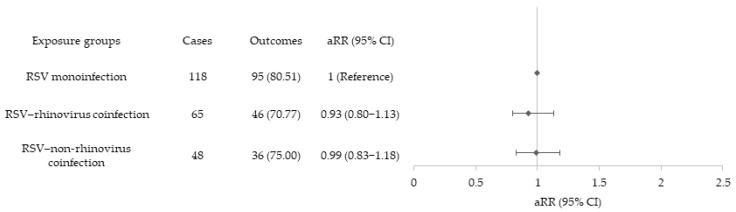
Multivariable Poisson regression analysis with robust (sandwich) variance estimator evaluating the association between exposure (RSV monoinfection vs. RSV–rhinovirus coinfection vs. RSV–non–rhinovirus coinfection) and severe outcome. Adjusted risk ratios (aRRs) with 95% confidence intervals (95% CIs) were adjusted for age, sex, and comorbidities.

After adjustment for relevant covariates (i.e., age group, sex, and presence of comorbidities), neither RSV–rhinovirus coinfection (aRR = 0.95; 95% CI = 0.80–1.13) nor RSV–non-rhinovirus coinfection (aRR = 0.99; 95% CI = 0.83–1.18) was associated with an increased risk of severe disease compared with RSV monoinfection (Figure 1).

No significant associations with severe outcome were observed for age group (1 month–<1 year vs. <1 month: aRR = 1.00; 95% CI = 0.73–1.38, and 1–5 year vs. <1 month: aRR = 0.72; 95% CI = 0.49–1.05) or sex (Male vs. Female: aRR = 1.04; 95% CI = 0.91–1.20). The presence of comorbidities was associated with a higher risk of severe outcome (aRR = 1.22; 95% CI = 1.06–1.41) (Table 5).

## 4. Discussion

In this multicenter retrospective cohort study of children aged ≤5 years hospitalized with RSV infection, we found that the presence of respiratory viral coinfection was not independently associated with increased disease severity. This finding was consistent across both RSV–rhinovirus and RSV–non-rhinovirus coinfections, suggesting that viral coinfection per se—including the most common pairing with rhinovirus—does not confer an additional risk of severe RSV disease among hospitalized children. Overall, our results align with evidence from both high-income and low- and middle-income settings showing no consistent association between RSV coinfection and clinical severity [24,25].

The lack of an independent association between RSV–rhinovirus coinfection and severe outcomes is consistent with recent cohort studies reporting comparable or even lower severity relative to RSV monoinfection [26,27]. Several population-level and mechanistic studies have proposed viral interference as a potential explanation. Surveillance analyses have demonstrated asynchronous circulation patterns between RSV and rhinovirus, with lower-than-expected rates of simultaneous detection, suggesting biologically meaningful virus–virus interactions [28,29]. In line with this, birth cohort data have shown a negative association between RSV and rhinovirus detection, supporting the hypothesis that infection with one virus may transiently reduce susceptibility to the other [16]. Experimental and translational studies further indicate that rhinovirus may induce robust innate immune responses, including interferon-mediated pathways, which may suppress RSV replication and mitigate lower respiratory tract involvement [13,30]. At the same time, clinical studies on RSV–rhinovirus coinfection have reported heterogeneous findings, with outcomes ranging from comparable or attenuated disease to increased severity across different populations and study designs [17]. Together, these data support the concept that RSV–rhinovirus interactions are complex and context-dependent, rather than uniformly protective.

In contrast, previous studies have reported increased disease severity in children coinfected with RSV and specific non-rhinovirus respiratory pathogens, including influenza, adenovirus, and human metapneumovirus, with higher risks of PICU admission, need for advanced respiratory support, and prolonged hospitalization [3,31]. Recent meta-analyses have emphasized that adverse outcomes are more likely driven by specific viral pairings rather than coinfection status alone [32,33]. Viruses such as influenza and human metapneumovirus share tropism for the lower respiratory tract and may amplify RSV-related lung injury through additive inflammatory mechanisms, providing biological plausibility for increased severity in selected RSV–non-rhinovirus coinfections [31]. In our cohort, we did not observe such associations, which may reflect the relatively small number of children coinfected with these specific pathogens. In our cohort, the longer hospitalization observed among children with RSV–non-rhinovirus coinfections should be interpreted with caution. This group comprised a heterogeneous mix of respiratory viruses, including adenovirus, influenza, and metapneumovirus, some of which have been previously associated with more severe lower respiratory tract disease. However, the study was not powered to evaluate the effect of individual viral pathogens, and residual confounding by host factors, including age and clinical presentation, cannot be excluded. Conversely, the shorter hospital stay observed in children with RSV–rhinovirus coinfection may reflect both the older age distribution of this group and potential virus–virus interactions. Similar findings have been reported in prior studies, where RSV–rhinovirus coinfection was associated with comparable or milder clinical courses compared with RSV monoinfection, although results remain inconsistent across settings [31,34].

Age distribution differed across exposure groups, with RSV–rhinovirus coinfection more common among children aged 1–5 years, whereas RSV monoinfection and RSV–non-rhinovirus coinfection predominated in younger infants. This pattern mirrors prior evidence showing that RSV–rhinovirus coinfections are more frequent in older children [26].

Given that young age is one of the strongest predictors of RSV severity, this age imbalance likely contributed the high burden of severe disease observed among infants hospitalized with RSV monoinfection, independent of coinfection status.

This study has several strengths. It includes a contemporary multicenter cohort evaluated using multiplex PCR diagnostics, reflecting current clinical practice. The use of a clinically meaningful composite severity outcome captures both disease severity and healthcare resource utilization. Most notably, the analytic separation of RSV–rhinovirus and RSV–non-rhinovirus coinfections align with emerging evidence and improves clinical interpretability. Adjustment for major confounders, including age, and comorbidities, further strengthens the robustness of the findings.

This study has several limitations that should be considered when interpreting the findings. The retrospective design introduces potential residual confounding and relies on routinely collected clinical data. PCR-based detection cannot distinguish active infection from prolonged viral shedding, particularly for rhinovirus, potentially leading to misclassification of coinfection status. Viral load, timing of infection, and bacterial coinfection were not systematically assessed and may have influenced outcomes. In particular, recent prospective data suggest that bacterial coinfections may be associated with increased disease severity, including higher ICU admission rates and longer hospitalization, although this association may in part reflect testing bias toward more severe cases [35]. Selection bias is possible, as multiplex testing may have been preferentially performed in younger or more severely ill children. In addition, although RSV immunization with nirsevimab and the maternal vaccine was introduced in Italy during the study period, its implementation was heterogeneous across regions, and individual-level data on immunization status were not consistently available. As a result, we were unable to assess the potential impact of RSV immunization on disease severity or coinfection patterns. This may have influenced the observed clinical profile of hospitalized children and should be addressed in future prospective studies. Finally, the study was conducted in tertiary-care hospitals, which may limit generalizability to outpatient or community settings.

## 5. Conclusions

In this study, most RSV coinfections were attributable to rhinovirus. Viral coinfection, including RSV–rhinovirus, was not independently associated with increased disease severity compared with RSV monoinfection. These findings support a more nuanced, pathogen-specific interpretation of multiplex PCR results, highlighting the importance of distinguishing RSV–rhinovirus from other viral coinfections. Further studies are needed to better define the clinical impact of specific viral coinfections.

## Figures and Tables

**Table 1:** Acute respiratory tract infection (ARTI) admissions at Pietro Barilla Children’s Hospital (Parma, Italy) and Bambino Gesù Children’s Hospital (Rome, Italy) during the study period (from 1 September 2022 to 30 April 2025), by exposure group (RSV monoinfection vs. RSV–rhinovirus coinfection vs. RSV–non-rhinovirus coinfection) and detected virus(es).

Exposure Group	Number of Cases (%)
RSV monoinfection	118 (51.08)
RSV–rhinovirus coinfection	65 (28.14)
RSV–non-rhinovirus coinfection	48 (20.78)
Adenovirus	25 (52.08)
Coronavirus (non-SARS-CoV-2)	22 (45.83)
Influenza	11 (22.92)
Parainfluenza	7 (14.58)
Metapneumovirus	7 (14.58)
SARS-CoV-2	6 (12.50)

**Table 2:** Sociodemographic and clinical characteristics by exposure group (RSV monoinfection vs. RSV–rhinovirus coinfection vs. RSV–non-rhinovirus coinfection).

	RSV Monoinfection *N* = 118		RSV–Rhinovirus Coinfection *N* = 65		RSV–Non-Rhinovirus Coinfection *N* = 48		*p*-Value
	*N*	%	*N*	%	*N*	%	
Male	70	59.32	38	58.46	24	50.00	0.529
Age							
<1 month	6	5.08	1	1.54	3	6.25	0.003
1 month–<1 year	93	78.81	37	56.92	32	66.67	
1–5 years	19	16.10	27	41.54	13	27.08	
Ethnicity							
Caucasian	97	82.20	55	84.62	42	87.50	0.880
African	6	5.08	3	4.62	0	0.00	
Asian	4	3.39	1	1.54	2	4.17	
Middle Eastern	9	7.63	5	7.69	4	8.33	
Hispanic	2	1.69	1	1.54	0	0.00	
Prematurity	9	7.63	4	6.15	4	8.33	0.945
Late preterm	6	66.67	2	50.00	3	75.00	0.891
Very preterm	2	22.22	2	50.00	1	25.00	
Extremely preterm	1	11.11	0	0.00	0	0.00	
Low birth weight	10	8.47	3	4.62	1	2.08	0.288
At least a comorbidity	28	23.73	16	24.62	6	12.50	0.222
Chronic respiratory diseases	5	4.24	2	3.08	1	2.08	0.899
Chronic cardiac diseases	3	2.54	5	7.69	1	2.08	0.206
Chronic neurological diseases	3	2.54	2	3.08	0	0.00	0.710
Chronic renal diseases	1	0.85	1	1.54	0	0.00	1.000
Other chronic diseases	14	11.86	10	15.38	2	4.17	0.168

RSV: Respiratory syncytial virus.

**Table 3:** Clinical symptoms at admission by exposure group (RSV monoinfection vs. RSV–rhinovirus coinfection vs. RSV–non-rhinovirus coinfection).

	RSV Monoinfection *N* = 118		RSV–Rhinovirus Coinfection *N* = 65		RSV–Non-Rhinovirus Coinfection *N* = 48		*p*-Value
	*N*	%	*N*	%	*N*	%	
Fever	62	52.54	39	60.00	28	58.33	0.578
Rhinitis	48	40.68	16	24.62	22	45.83	0.034
Cough	93	78.81	49	75.38	36	75.00	0.809
Pharyngitis	25	21.19	17	26.15	10	20.83	0.708
Loss of appetite	52	44.07	18	27.69	22	45.83	0.061
Asthenia	2	1.69	1	1.54	0	0.00	1.000
Gastrointestinal symptoms	10	8.47	13	20.00	8	16.67	0.069
Conjunctivitis	2	1.69	3	4.62	2	4.17	0.461
Other *	65	55.08	35	53.85	25	52.08	0.939

RSV: Respiratory syncytial virus; * Other symptoms included dyspnea, distress, tachypnea, polypnea, apnea, retractions, abdominal breathing, oliguria, eye redness, ear pain, otitis, stye, dermatitis, rash, xerosis, vomiting, hypotonia, hypoactivity, fever, hoarseness, chest pain, hand–foot–mouth disease, and crying.

**Table 4:** Disease severity by exposure group (RSV monoinfection vs. RSV–rhinovirus coinfection vs. RSV–non-rhinovirus coinfection).

	RSV Monoinfection *N* = 118		RSV–Rhinovirus Coinfection *N* = 65		RSV–Non-Rhinovirus Coinfection *N* = 48		*p*-Value
	*N*	%	*N*	%	*N*	%	
ICU admission	6	5.08	4	6.15	5	10.42	0.464
Respiratory support	95	80.51	46	70.77	36	75.00	0.315
Oxygen	95	100.00	46	100.00	36	100.00	-
Non-invasive	9	9.47	6	13.04	5	13.89	0.706
Invasive	3	3.16	0	0.00	1	2.78	0.652
Hemodynamic support	1	0.85	0	0.00	0	0.00	1.000
Severe disease	95	80.51	46	70.77	36	75.00	0.315
Hospitalization length							
≤1 week	87	73.73	58	89.23	30	62.50	0.003
1 week–≤1 month	31	26.27	7	10.77	17	35.42	
>1 month	0	0.00	0	0.00	1	2.08	

RSV: Respiratory syncytial virus; ICU: Intensive care unit.

**Table 5:** Multivariable Poisson regression analysis with robust (sandwich) variance estimator evaluating the association between exposure (RSV monoinfection vs. RSV–rhinovirus coinfection vs. RSV–non-rhinovirus coinfection) and severe outcome.

	*N*	Severe Disease	aRR (95% CI) *
Age			
<1 month	10	8 (80.00)	1 (reference)
1 month–<1 year	162	133 (82.10)	1.00 (0.73–1.38)
1–5 years	59	36 (61.02)	0.72 (0.49–1.05)
Sex			
Female	99	75 (75.76)	1 (reference)
Male	132	102 (77.27)	1.04 (0.91–1.20)
Comorbidities			
No	181	135 (74.59)	1 (reference)
Yes	50	42 (84.00)	1.22 (1.06–1.41)

* Adjusted risk ratios (aRRs) with 95% confidence intervals (95% CIs) were adjusted for age, sex, and comorbidities.

## Data Availability

Available upon request.

## Supplementary Materials

The following supporting information can be downloaded at: https://www.mdpi.com/article/10.3390/v18040451/s1, Supplemental Figure S1. Flowchart of inclusion and exclusion criteria; Supplemental Figure S2. Number of cases over time (from 1 September 2022 to 30 April 2025) by exposure (RSV monoinfection vs. RSV–rhinovirus coinfection vs. RSV–non-rhinovirus coinfection); Supplemental Figure S3. Acute respiratory tract infection (ARTI) admissions at Pietro Barilla Children’s Hospital (Parma, Italy) and Bambino Gesù Children’s Hospital (Rome, Italy) during the study period (from 1 September 2022 to 30 April 2025), by detected virus(es).

## Abbreviations

The following abbreviations are used in this manuscript:

RSV	Respiratory syncytial virus
URTI	upper respiratory tract infection
LRTI	lower respiratory tract infection
ARTI	acute respiratory tract infection
PICU	pediatric intensive care unit
PCR	polymerase chain reaction
aRRs	adjust risk ratios
95% CIs	95% confidence intervals
