# Supplementary material for: Disease Severity of Respiratory Syncytial Virus Infection in Hospitalized Children
Source: Viruses. 2026 Apr 9;18(4):451. doi: 10.3390/v18040451 (PMC13120199; doi:10.3390/v18040451)

**Supplemental materials**

Disease severity of Respiratory Syncytial Virus monoinfection and with respiratory viral coinfection in hospitalized children

*Supplemental materials list*

**Supplemental Figure S1.** Flowchart of inclusion and exclusion criteria.

**Supplemental Figure S2.** Number of cases over time (from 1 September 2022 to 30 April 2025) by exposure (RSV monoinfection vs. RSV–rhinovirus coinfection vs. RSV–non-rhinovirus coinfection).

**Supplemental Figure S3.** Acute respiratory tract infection (ARTI) admissions at Pietro Barilla Children's Hospital (Parma, Italy) and Bambino Gesù Children's Hospital (Rome, Italy) during the study period (from 1 September 2022 to 30 April 2025), by detected virus(es).

***Supplemental figure S1***. Flowchart of inclusion and exclusion criteria.

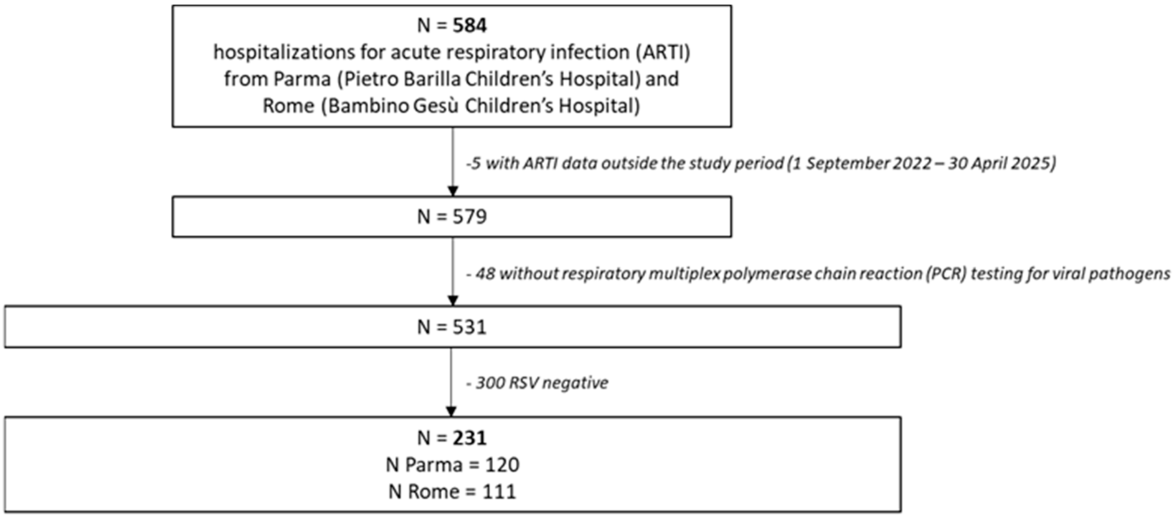

***Supplemental Figure S2.*** Number of cases over time (from 1 September 2022 to 30 April 2025) by exposure (RSV monoinfection vs. RSV–rhinovirus coinfection vs. RSV–non-rhinovirus coinfection).

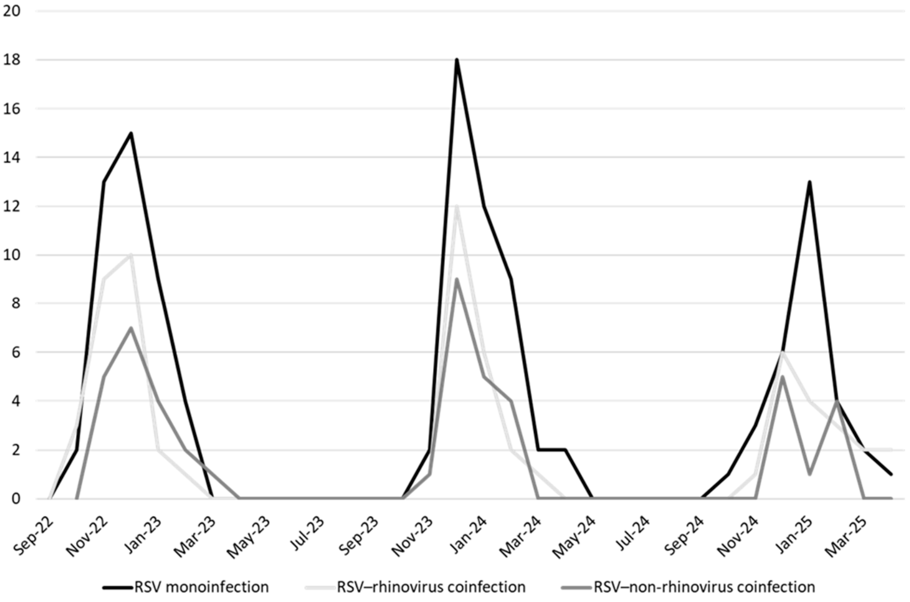

***Supplemental figure S3***. Acute respiratory tract infection (ARTI) admissions at Pietro Barilla Children's Hospital (Parma, Italy) and Bambino Gesù Children's Hospital (Rome, Italy) during the study period (from 1 September 2022 to 30 April 2025), by detected virus(es).

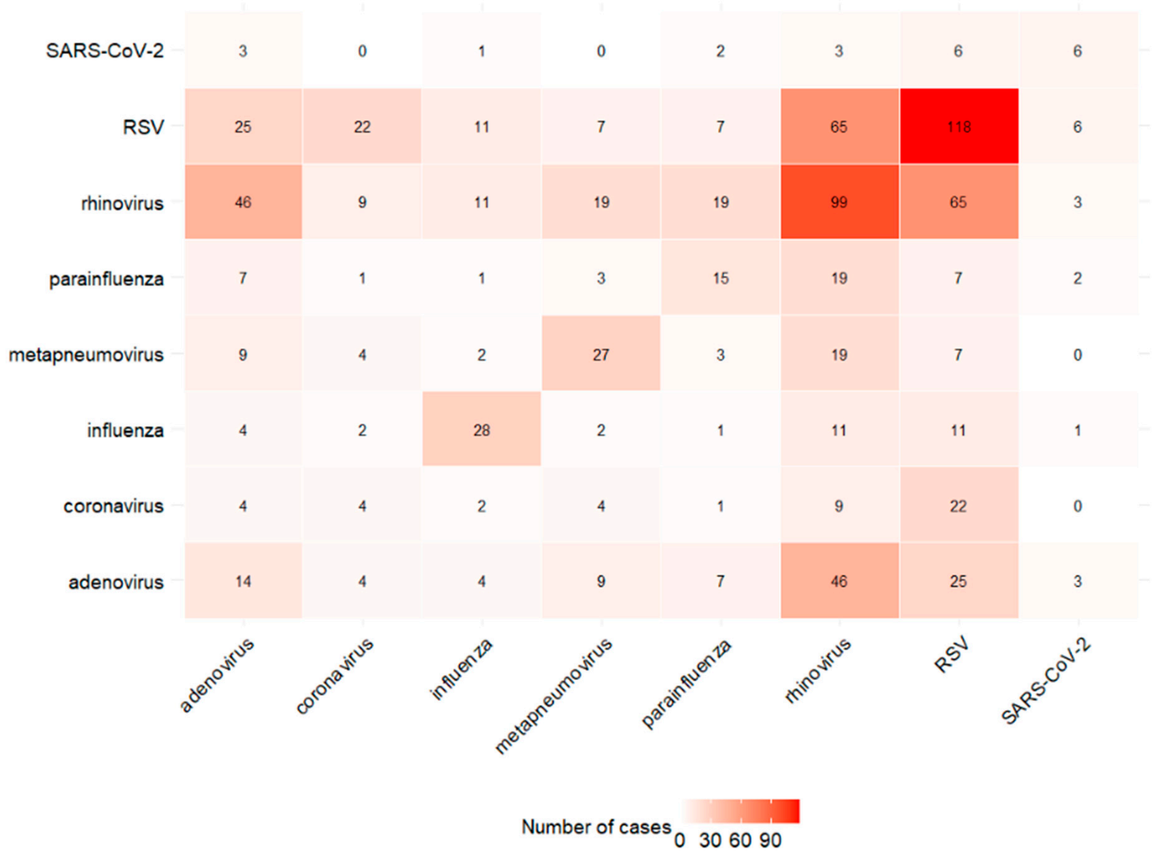

Supplement: Supplementary file 1 [file viruses-18-00451-s001.zip › viruses-4184592-supplementary.pdf]
